# Relationship between Systemic Inflammation and Delayed-Type Hypersensitivity Response to Candida Antigen in Older Adults

**DOI:** 10.1371/journal.pone.0036403

**Published:** 2012-05-02

**Authors:** Brandt D. Pence, Thomas W. Lowder, K. Todd Keylock, Victoria J. Vieira Potter, Marc D. Cook, Edward McAuley, Jeffrey A. Woods

**Affiliations:** 1 Department of Kinesiology and Community Health, University of Illinois at Urbana-Champaign, Urbana, Illinois, United States of America; 2 Integrative Immunology and Behavior Program, University of Illinois at Urbana-Champaign, Urbana, Illinois, United States of America; 3 Division of Nutritional Sciences, University of Illinois at Urbana-Champaign, Urbana, Illinois, United States of America; 4 Beckman Institute for Advanced Science and Technology, University of Illinois at Urbana-Champaign, Urbana, Illinois, United States of America; 5 Department of Psychology, University of Illinois at Urbana-Champaign, Urbana, Illinois, United States of America; 6 Department of Pathology, University of Illinois at Urbana-Champaign, Urbana, Illinois, United States of America; University of Nebraska Medical Center, United States of America

## Abstract

Research has shown that aging is associated with increased systemic inflammation as well as a reduction in the strength of immune responses. However, little evidence exists linking the decrease in cell-mediated immunity in older adults with other health parameters. We sought to examine the relationship between cell-mediated immunity as measured in vivo by the delayed-type hypersensitivity (DTH) response to candida antigen and demographic and physiological variables in older (65–80 y.o.) adults. Candida antigen response was not related to gender or obesity, or to a number of other physiological variables including fitness and body composition. However, positive responders had significantly lower serum C-reactive protein levels (CRP, p<0.05) vs. non-responders. Furthermore, subjects with CRP<4.75 mg•L^−1^ had greater odds of developing a positive response compared to those with CRP>4.75 mg•L^−1^. Therefore, positive responses to candida antigen in older adults appears to be related to lower levels of systemic inflammation.

## Introduction

A large amount of evidence indicates that chronological aging is associated with immune system dysfunction. This progressive loss of immune function is termed immunosenescence [1] and includes changes in function in innate, humoral and cell-mediated immunity. Aging is associated with a decline in the number and function of circulating dendritic cells [2] as well as a decrease in diversity and antigen-specificity of circulating B cells [3]. Changes in immune function of this type likely play a major role in the general decrease in immune response to vaccination in older adults, as these individuals are less likely to develop or maintain protective antibody responses against influenza virus [4] and hepatitis A and B viruses [5], among others.

In addition to the evidence for a progressive age-related decrease in humoral immune responses to vaccination, there is a considerable body of literature demonstrating that cell-mediated immune responses are impaired in elderly individuals. In this population, circulating numbers of naïve CD8+ cytotoxic T cells are reduced with a concordant increase in memory T cell numbers, thereby decreasing the diversity of T-cell receptors (TCR) available for pathogen defense [6]. Impaired T cell responses have been associated with high morbidity and mortality in older adults [7]. One study showed an association between non-reaction to delayed-type hypersensitivity (DTH), a global measure of cell-mediated immunity [8] and an increase in incidence of hospital mortality in surgical patients [9], and declining DTH responses have also been shown to correlated with increased disease burden in older adults [10], but little additional evidence for an association between DTH responses and other health parameters in older adults currently exists.

Aging is also known to negatively affect innate immunity and inflammation. Increased circulating cytokines including tumor necrosis factor-α and interleukin-6 as well as increased circulating levels of acute phase protein C-reactive protein (CRP) have been demonstrated in elderly individuals and associated with impaired inflammatory responses to vaccination [1], [11]. Increased inflammation, along with impairment in immune responses, is associated with an immune risk phenotype that has been shown to be correlated with mortality in older adults [7].

As noted above, circulating inflammatory markers are known to be associated with poor vaccine responses in the elderly [11]. Additionally, in the aged population, obesity [12] and moderate physical activity [13] are known to be pro- and anti-inflammatory, respectively, and thus may regulate inflammation and, by extension, immune responses in an elderly population. Thus, we examined the relationships between inflammation, body composition, and physical fitness and association with cell-mediated immunity using the DTH response to intradermal injection of antigen purified from *Candida albicans*. We hypothesized that a positive DTH response to candida antigen would be related to higher levels of baseline physical fitness, to lack of obesity, and to reduced inflammation in these subjects.

## Results

### Differences in DTH Response Due to Classification

**Figure 1:**
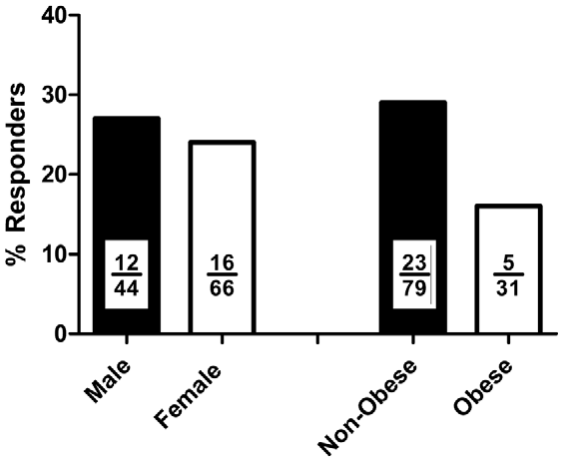
Percentage of subjects responding to DTH induced by candida antigen. Bar insets represent number of positive responders (numerator) over total number of subjects (denominator) in each group.

**Figure 2:**
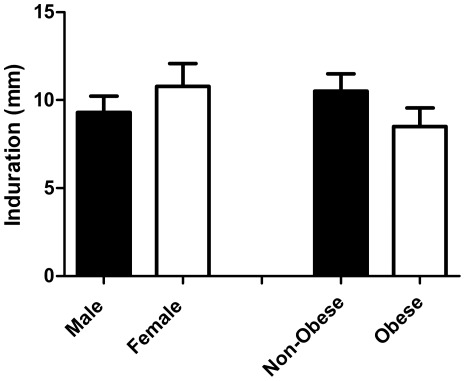
Magnitude of DTH response among positive responders to candida antigen. Values are mean ± SEM.

Pearson chi-squared statistics were calculated to determine if the percentages of responders to DTH induced by candida antigen were different when subjects were classified according to distinguishing characteristics. The percentage of males that responded to the DTH test was not different than the percentage of females that responded to the test (Χ^2^_(df = 1)_ = 0.128, *p* = 0.712, Figure 1). Likewise, the percentage of obese (BMI≥30) subjects who responded to the DTH test was not different when compared to the percentage of non-obese subjects (BMI<30) who responded to the DTH test (Χ^2^_(df = 1)_ = 1.978, *p* = 0.160, Figure 2). Analysis of classification differences in only the positive responders revealed no difference in mean induration between males and females (t_(df = 26)_ = 0.868, *p* = 0.393, Figure 2) or between obese and non-obese subjects (t_(df = 26)_ = 0.904, *p* = 0.375, Figure 2). With no significant differences in either response rate or mean response to induction of DTH in the subjects, we collapsed all subjects into responders and non-responders groups and examined the differences in anthropometric characteristics between groups.

### Anthropometric Differences between Responders and Non-Responders to Candida Antigen

**Figure 3:**
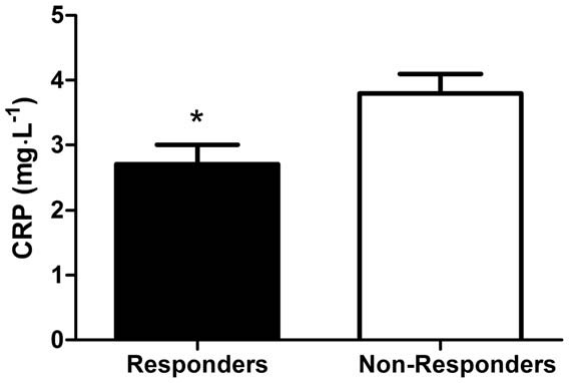
CRP values among responders and non-responders to DTH induced by candida antigen. *Significantly different compared to non-responders (p<0.05). Values are mean ± SEM.

Independent samples t-tests were performed to assess the differences in anthropometric characteristics between responders and non-responders to DTH induced by injection of candida antigen. We assessed between-group differences in age as well as in markers of body composition (body mass index, body weight, body fat percentage) and cardiovascular fitness (VO_2_ peak, resting heart rate). We also assessed between group differences in number of anti-inflammatory medicines taken as well as in basal levels of systemic inflammation (serum C-reactive protein). Table 1 shows the differences in these characteristics between responders and non-responders. No difference (*p*>0.05) in mean age, VO_2_ peak, body mass index (BMI), body weight, body fat percentage, resting heart rate (RHR), or anti-inflammatory medication usage were found between responders and non-responders to DTH (Table 1). However, serum C-reactive protein (CRP) levels were significantly lower in responders (2.7±0.3 mg·L^−1^) than in non-responders (3.8±0.3 mg·L^−1^, t_(df = 72.077)_ = 2.583, *p* = 0.012, Figure 3). Therefore, we sought to further characterize the relationship between serum CRP levels and DTH response to candida antigen.

**Table 1:** Baseline characteristics.

	Responders	Non-Responders	*p*
	(N = 28)	(N = 82)	
**Age (yr)**	69.7±1.1	69.9±0.6	*0.898*
	(68.0, 62.0–83.0)	(70.0, 60–83.0)	
**VO_2_ Peak (ml·kg^−1^·min^−1^)**	19.7±0.8	20.0±0.4	*0.758*
	(19.1, 12.7–31.5)	(20.2, 19.3–30.4)	
**BMI (kg·m^−2^)**	26.8±0.8	28.0±0.5	*0.228*
	(27.0, 19.0–36.0)	(27.0, 20.0–41.0)	
**Weight (kg)**	76.5±2.9	78.0±1.7	*0.655*
	(75.3, 46.4–112.1)	(74.8, 50.5–113.5)	
**Body Fat (%)**	33.0±1.2	34.0±0.9	*0.540*
	(33.4, 21.4–45.0)	(33.7, 16.4–52.9)	
**Resting Heart Rate (bpm)**	72.4±2.2	75.4±1.5	*0.310*
	(75.0, 51.0–102.0)	(73.0, 54.0–120.0)	
**Anti-Inflammatory Meds (#)**	0.6±0.1	0.8±0.1	*0.455*
	(1.0, 0.0–2.0)	(1.0, 0.0–2.0)	

Values shown are mean ± SEM. Parenthetical values shown are (median, range). yr: years of age. bpm: beats per minute. #: total number of medications.

### Relationship between Serum CRP and DTH Response to Candida Antigen

In order to assess the relationship between serum CRP and DTH response to candida antigen, we collapsed responders and non-responders into a single group, broke the resulting data into quartiles based on serum CRP level, and performed multinomial logistic regression to establish the odds ratios for the development of a positive response, comparing each quartile to the highest quartile (serum CRP≥4.76 mg·L^−1^). Those in the 2nd quartile (serum CRP 1.87–3.08 mg·L^−1^) had 6.94× greater odds (*p* = 0.020) of developing a positive response compared to subjects in the highest quartile (Table 2). Subjects in the 1st (serum CRP 0–1.86 mg·L^−1^) and 3rd quartile (serum CRP 3.09–4.75 mg·L^−1^) had, respectively, 4.38× (p = 0.085) and 5.00× (*p* = 0.057) greater odds of developing a positive response compared to those in the highest quartile, although these ratios were not statistically significant (Table 2). Additionally, the percentage of responders was relatively consistent between the 1^st^–3^rd^ quartiles, while only 2 of the 28 responders (7.1%) came from the 4^th^ and highest quartile (Table 2). Therefore, we concluded that serum CRP>4.75 mg·L^−1^ is related to impairment in the ability of an individual to mount a DTH response to candida antigen, a finding that may indicate a general dysregulation of cell-mediated immunity in this group.

**Table 2:** Odds ratios for positive DTH response by CRP level.

CRP Quartile	OR (95% CI)	*p*	Responders (%)
**1st (0.00–1.86 mg·L^−1^)**	4.38 (0.82–23.42)	*0.085*	8 (28.6%)
**2nd (1.87–3.08 mg·L^−1^)**	6.94 (1.35–35.61)	*0.020* *	10 (35.7%)
**3rd (3.09–4.75 mg·L^−1^)**	5.00 (0.95–26.23)	*0.057*	8 (28.6%)
**4th (4.76+ mg·L^−1^)**	1.00	—	2 (7.1%)

* Significantly increased odds of developing a positive DTH response (p<0.05). CI, confidence interval. % Responders represents percentage of total responders (N = 28) per group.

### Characteristics of Positive Responders

We then sought to determine if any of our predictor variables had a significant relationship with magnitude of the DTH induration response to candida antigen in those subjects who had a positive response to the test. We began with an analysis of the correlations between predictor variables and induration response in these subjects. Magnitude of induration was not significantly correlated with any of the predictor variables in positive responders, including CRP (Table 3). Among predictor variables, age was significantly correlated to both body fat percentage and BMI, while BMI was significantly correlated to body weight and body fat percentage (Table 3), findings that were not surprising. This suggested that the magnitude of the DTH response to candida antigen among positive responders was not related to our predictor variables.

**Table 3:** Correlation between DTH response to candida antigen and predictor variables in positive responders.

	Age	VO_2_ Peak	Weight	BMI	Body Fat	RHR	CRP	Meds	DTH
	(yr)	(ml·kg^−1^·min^−1^)	(kg)	(kg·m^−2^)	(%)	(bpm)	(mg·L^−1^)	(#)	(mm)
**Age**	1.000	0.133	−0.345	−0.436	−0.599	−0.150	−0.156	−0.051	−0.198
*p*	—	*0.499*	*0.072*	*0.020* *	*0.001* *	*0.447*	*0.427*	*0.796*	*0.312*
**VO_2_ Peak**		1.000	0.142	0.002	0.032	−0.277	−0.214	−0.078	0.217
*P*		—	*0.472*	*0.992*	*0.870*	*0.154*	*0.273*	*0.694*	*0.267*
**Weight**			1.000	0.912	0.190	−0.027	0.136	−0.118	0.046
*P*			—	*0.000* *	*0.332*	*0.891*	*0.492*	*0.551*	*0.818*
**BMI**				1.000	0.431	0.051	0.216	−0.166	0.152
*P*				—	*0.022* *	*0.798*	*0.271*	*0.398*	*0.441*
**Body Fat**					1.000	0.149	0.196	−0.013	0.231
*P*					—	*0.448*	*0.317*	*0.948*	*0.238*
**RHR**						1.000	−0.084	−0.027	−0.005
*P*						—	*0.670*	*0.892*	*0.981*
**CRP**							1.000	−0.183	−0.028
*P*							—	*0.351*	*0.887*
**Meds**								1.000	−0.213
*p*								—	*0.276*

* Significant correlation between variables (p<0.05). VO_2_, volume of oxygen consumption; BMI, body mass index; RHR, resting heart rate; CRP, C-reactive protein; Meds, anti-inflammatory medications; DTH, delayed-type hypersensitivity; bpm, beats per minute.

## Discussion

Aging is associated with a decrease in the ability of the immune system to respond effectively to various stimuli, a phenomenon which has been termed immunosenescence [1]. Despite much research in this area, little is known about the mechanisms which underlie this state. Paradoxically, while immune status is impaired in the elderly, there also exists a state of chronic low-level inflammation in the same population which includes higher-than-normal circulating levels of pro-inflammatory cytokines when compared to younger subjects [14]. Additionally, obesity [12], gender [15], and physical activity status [13] are known to impact inflammation and immunity in older adults. Thus, we sought to examine the roles of inflammation, body composition, gender, and fitness on an *in vivo* marker of cell-mediated immunity - the delayed-type hypersensitivity (DTH) response to antigen purified from *Candida albicans*.

There was no impact of gender on the proportion of subjects who responded to candida antigen injection. This was not surprising, as all women in the study were post-menopausal, and removal of the estrogen stimulus abrogates the enhanced immunity seen in females when compared to males [15]. More interestingly, a higher proportion of non-obese subjects responded to candida antigen, although this result was non-significant. This finding suggests that further investigation along this line may be of interest.

The main finding in this study was that responders to candida antigen had lower systemic inflammation as measured by serum CRP levels when compared to subjects who did not respond to candida antigen. Due to the unbalanced numbers of subjects in each group (28 responders, 82 non-responders), we also examined the rates of response by quartile of CRP. Those subjects in the lower 3 quartiles of CRP (≤4.75 mg•L^−1^) had a 4.38–6.94× higher likelihood of responding positively to candida antigen when compared to those in the highest quartile (>4.75 mg•L^−1^) of CRP level. Because 10 subjects in the second quartile had positive responses compared to 8 subjects in each of quartiles 1 and 3, only the second quartile had significantly greater odds of developing a positive response compared to the fourth quartile. However, the odds ratios approached significance for quartiles 1 and 3 (p<0.10). Interestingly, CRP levels were not related to the magnitude of response in individuals who did respond to candida antigen, suggesting that systemic inflammation in those who do mount a successful T cell response to candida antigen may not play a role in the strength of that response.

The results of this study suggest that higher systemic inflammation may be associated with poorer immune responses. This has been previously demonstrated in vaccine studies in which subjects with increased inflammation have reduced antibody responses to vaccination [11]. Further, the age-related increase in circulating inflammatory markers is related to the development of other diseases and disorders that are associated with aging, including Alzheimer's disease, type 2 diabetes, coronary heart disease, and others [16], [17], [18], [19]. However, to our knowledge this study is the first to link age-related systemic increases in CRP, a clinically-important measure of inflammation [20] to a decreased T cell-mediated immune response to a skin test antigen.

There are several potential limitations to the study described herein. First, our subject population excluded a number of individuals, primarily for the presence of conditions which might affect inflammatory or immune responses. These include autoimmune and inflammatory diseases, smoking (within 10 years prior to the study), use of strong anti-inflammatory medications, and a number of others. This was done primarily to reduce the number of confounding factors which might affect the primary outcomes in the ImFIT trial. However, such exclusions may limit the generalizability of the results present here in the population at large.

Another major limitation is the use of only one measure of cell-mediated immune response, namely the DTH response to candida antigen. As there may be a number of mitigating factors which impact the odds of a positive response in any individual (including lack of prior exposure to the antigen), additional work is necessary to be able to make conclusions about the link between inflammation and global cell-mediated immune responses. These studies should use a number of additional DTH measures in concert and could be further strengthened by the examination of other circulating inflammatory markers as well as *in vitro* measures of cell-mediated immunity.

Further study is needed to test potential mechanisms for this observation. A number of theories as to the cause of age-related immunosenescence have been postulated in recent years. Perhaps the most well-established is the increase in cytomegalovirus (CMV)-specific senescent memory T cells in older adults [21]. CMV seropositivity and a T cell CD4∶CD8 ratio of <1 are the chief markers in the immune risk profile which predicts mortality in adults >85 years of age [1], [22]. It has been shown that aging is associated with a reduction in circulating naïve CD8+ T cells along with a concomitant increase in memory T cells, a phenomenon that reduces the diversity of T cell antigen receptors (TCR) in the circulating T cell pool and is thought to be the driving force behind reductions in T cell function with novel infection in older adults [1], [6]. Whether this observation is related to the increase in inflammation seen in aging (“inflammaging” [14]) is unknown, but our findings represent a potential link between inflammaging and immunosenescence.

Of major interest is the question of whether increased inflammation in itself is a driver of immunosenescence, or whether immunosenescence and inflammaging are both symptomatic of the same underlying cause. One potential link, as stated above, is CMV status. Frequent reactivation of chronic viral infections such as CMV are thought to induce immunosenescence and, speculatively, may also induce frequent activation of viral-specific T cells and increase circulating inflammation. Of course, associative studies such as this cannot prove causality, and further mechanistic studies are need to demonstrably link, whether directly or indirectly, the decreased T cell function and the increased systemic inflammation seen our aged subjects.

Of additional interest is the study of therapies which may reduce systemic inflammation and, potentially, improve cell-mediated immune responses in older adults. A variety of potential strategies ranging from pharmaceutical to nutritional in nature could be conceived that may have some benefit in improving immune responses. Previous research in our laboratory demonstrates that exercise decreases inflammation [23], [24] and improves immunity [25], [26], [27], [28] in older adults as well as in aged mice. A previous study has shown that exercise training can reverse the age-associated decrease in delayed-type hypersensitivity to the novel antigen keyhole limpet hemocyanin [29]; however, inflammation was not assessed in this study. These results are in line with a number of other studies which have shown decreases in inflammation and improvements in immunity with exercise training. This literature has been addressed comprehensively in several recent reviews [13], [30], [31]. We suggest that physical exercise may be an ideal therapy to improve cell-mediated immunity in older adults, and that it may act by reducing systemic inflammation. Future studies will be necessary to characterize the relationship between inflammation and cell-mediated immunity in older adults and to test potential therapies for improving both measures, including exercise.

### Conclusion

We have found that older adults who are positive responders to a delayed-type hypersensitivity test have, on average, lower circulating levels of the inflammatory marker CRP than those who do not respond to the injection. Further, CRP levels >4.75 mg•L^−1^ are associated with an increased risk of non-responsiveness. However, no relationship existed between CRP and strength of response in the responder group. Future research is necessary to characterize the relationship between inflammation and cell-mediated immunity in older adults.

## Materials and Methods

### Subjects

**Figure 4:**
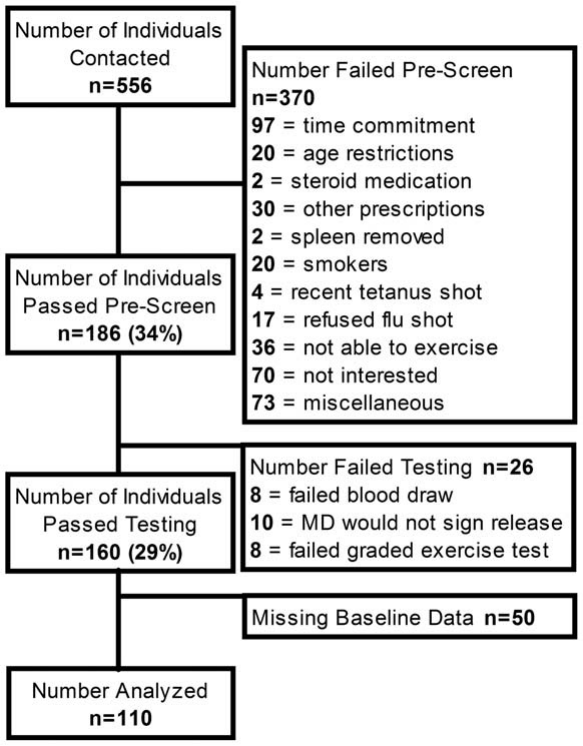
Immune Function Intervention Trial CONSORT diagram.

Subjects were recruited as part of the Immune Function Intervention Trial (ImFIT), a randomized clinical exercise intervention trial examining the impact of exercise on immunological parameters primarily including vaccine response, the results of which have been published elsewhere [32]. The cross-sectional analyses reported here were performed on baseline data collected as part of the ImFIT study in order to determine the factors which influence DTH response in sedentary older adults. Sedentary older adults (60–83 yr) were recruited from the Urbana/Champaign area (pop. 168,000) via advertisement in local media as well as public places including local senior citizen centers. Subjects included in the study must have been sedentary for at least 6 months prior to the beginning of the study. Exclusion criteria used in the telephone pre-screening included recent (<1 yr) history of cancer, inflammatory disease (such as asthma, autoimmune disease, etc.), chronic obstructive pulmonary disorder, uncontrolled diabetes mellitus, congestive heart failure, recent illness, recent vaccination, smoking (within 10 years prior to enrollment), or use of strong anti-inflammatory medications such as corticosteroids. Subjects not excluded by the telephone pre-screening reported to the laboratory for baseline fasting blood and graded exercise testing. Abnormal results from complete blood counts and comprehensive metabolic panels performed on the subjects resulted in exclusion from the study. Subjects who passed the screening phase were accepted into the study. Subjects with missing baseline candida antigen test results were excluded from analysis. In total, 110 of 161 subjects had complete data sets and were included for analysis. A CONSORT diagram (Figure 4) outlines the process of recruitment, screening, and testing.

### Graded Exercise Testing

Subjects reported to the laboratory to undergo a preliminary evaluation of cardiovascular fitness via a graded walking exercise test (GXT) to volitional fatigue. All GXT were performed under the supervision of a physician as required by the Institutional Review Board (IRB) at the University of Illinois for studies utilizing subjects from older populations. The initial treadmill speed was set according to each subject's brisk, comfortable walking speed and ranged from 3.2–6.4 km/hr. Grade, or in some cases speed, was increased every 2 minutes until volitional fatigue. An indirect calorimetry system (ParvoMedics, Sandy, UT) was utilized to measure volume of oxygen consumption (VO_2_), volume of carbon dioxide production (VCO_2_), and ventilation (VE) during the test. Respiratory exchange ratio and blood pressure were measured at the end of each stage, and heart rate was assessed continuously during the test by 12-lead electrocardiogram (ECG) monitoring. Subjects with abnormal responses to the GXT were excluded from the study and referred to their personal physician for follow-up. The highest VO_2_ attained during testing was recorded as VO_2_ peak and used in further analyses as an indicator of cardiovascular fitness. Baseline resting heart rate was also recorded and used as a further indicator of the fitness of subjects as this variable is easier to measure clinically.

### Body Composition

Body composition was measured by dual energy X-ray absorptiometry (DXA, Hologic QDR 4500A, Waltham, MA) according to manufacturer's instructions. Whole body scans were used to provide fat mass measures. Body weight and body mass index (BMI) were also recorded as supplementary measures of body composition.

### Delayed-Type Hypersensitivity to Candida Antigen

Intradermal injections (0.1 ml) of skin test antigen purified from *Candida albicans* (Candin, Allermed, San Diego, CA) were used to quantify the delayed-type hypersensitivity response (DTH) as a measure of strength of cell-mediated immunity in sedentary older adults. Induration >5 mm at 2 days post-injection was recorded as a positive response, and induration induced by the antigen was subtracted from that induced by a negative saline control, if any. Subjects were asked to refrain from use of anti-histamines and non-steroidal anti-inflammatory drugs for 4 days prior-to and 2 days after the injection. Baseline responders made up 25% (28/110) of the total subject pool, and the characteristics of these individuals were compared to non-responders to determine if differences in predictor variables including inflammatory status, cardiovascular fitness, and/or body composition might predict a positive response in this subject population. Percentage of responders in this study was somewhat lower than has been reported in this age group in studies using the multi-antigen injections [33], which may be partially explained by the greater induration (5 mm vs. 2 mm) necessary to be classified as a positive score in our study.

### C-Reactive Protein and Anti-Inflammatory Medications

Blood was drawn from subjects following an overnight fast and centrifuged at 1200×*g* for 15 minutes at 4°C. Following centrifugation, serum was removed and stored at −80°C until analysis. Serum C-reactive protein (CRP) was quantified as a measure of systemic inflammation via high-sensitivity enzyme-linked immunosorbant assay (ELISA) according to manufacturer's instructions (Diagnostic Automation, Calabasas, CA). Intra- and inter-assay coefficients of variation were 4.4% and 3.3% respectively. Anti-inflammatory medication use was also assessed from subject screening data. Number of anti-inflammatory medications (including aspirin, statins) was recorded and used in analyses as a potential predictor of DTH response.

### Statistical Analyses

All data were analyzed using SPSS 19 (IBM, Somers, NY). Gender and obesity between-group differences in percentages of DTH responders and non-responders were assessed using Pearson chi-squared statistics. Anthropometric differences in predictor variables between DTH responders and non-responders were determined using independent-samples Student's t-tests. For stratification of CRP levels, quartiles of serum CRP were analyzed for odds ratios of positive response to DTH using multinomial logistic regression. Among positive responders, potential predictor variables were correlated with each other and with magnitude of the DTH response using Pearson's bivariate correlations. An α of 0.05 was used to determine significance in all statistical tests, and all tests were two-tailed.
